# Retroperitoneal packing as part of damage control surgery in a Danish trauma centre – fast, effective, and cost-effective

**DOI:** 10.1186/1757-7241-16-4

**Published:** 2008-07-21

**Authors:** Allan Bach, Jørgen Bendix, Keld Hougaard, Erika Frischknecht Christensen

**Affiliations:** 1 Surgical Gastroenterological Department L, Aarhus University Hospital, Denmark; 2 Department of Pathology, Aarhus University Hospital, Denmark; 3 Orthopaedic Department E, Aarhus University Hospital, Denmark; 4 Department of Anaesthesia and Intensive Care, Aarhus University Hospital, Denmark

## Abstract

**Background:**

Retroperitoneal packing in patients with severe haemorrhage is a cornerstone of modern pelvic fracture management. However, few Danish trauma surgeons have experience with this procedure, and trauma audits show that many hesitate to perform the procedure, indicating a need for hands-on training for this simple and potentially lifesaving procedure.

**Materials and methods:**

During a six-month period, trauma surgeons were taught the retroperitoneal packing procedure using human corpses at the Department of Pathology at Aarhus University Hospital.

**Results:**

The course consisted of a 30 minute long single training session in retroperitoneal packing. Twenty-three sessions were held. Forty-two trauma surgeons (the entire staff at Aarhus Trauma Centre) and ten observers completed the course. Afterwards, all participants felt competent to perform the procedure.

**Conclusion:**

All 42 surgeons at our local trauma organisation learned a simple lifesaving operation within a short time period. In the 12 months following the completion of the course, 11 patients were treated with packing without any hesitation and with success. Damage control surgery with packing was cost-effectively implemented at our centre with great ease and rapidity.

## Introduction

Uncontrollable bleeding in patients with pelvic fracture is a well-known life-threatening complication [1].

Damage control surgery is a relatively new concept, and retroperitoneal packing has rarely been performed in Denmark. Since it is so rarely needed, most surgeons have limited experience with this procedure. Trauma audits within our organisation have shown that surgeons often hesitate or do not perform this procedure even when retroperitoneal packing is indicated.

Since retroperitoneal packing is a very simple and potentially lifesaving procedure, all surgeons who receive trauma patients should be able to perform it correctly and without delay. Still, as described in the 'Formula of Survival' concept [2,3], no recommended procedure will change a patient's outcome without training and effective implementation.

We were inspired by the Top Knife course in Bergen [4] and have chosen to train and teach our trauma surgeons and anaesthesiologists how to perform retroperitoneal packing on human corpses.

The purpose of this project was to design and implement a simple, hands-on, short training course for surgeons and anaesthesiologists and evaluate the impact of the course. Our success criterion was having all involved doctors trained within six months. We evaluated the course by asking the doctors about their approach to deciding on and performing retroperitoneal packing. Finally, we monitored the changes in the number of procedures performed after the course and the outcome of the trauma audits.

## Materials and methods

Hands-on training of retroperitoneal packing was performed on human corpses because the human anatomy differs too much from other animals, i.e., pigs, for them to be used for this procedure. The corpses were intended to undergo ordinary autopsy. The course took place at the Department of Pathology at Aarhus University Hospital, NBG, Denmark.

The Danish National Committee on Biomedical Research Ethics was contacted for permission to perform the procedure on the corpses, although this was not needed, as no living humans were involved in the study. Apart from packing swabs in the abdomen and removing them again, the procedure did not differ from an ordinary autopsy.

The sessions were directed by the head of abdominal trauma surgery and ran over a six month period from December 2005 until June 2006. The sessions were initially scheduled for 20 Mondays, but were extended by six more sessions during that period. The sessions took place on Mondays because most corpses were available this day, since no autopsies were performed during the weekend.

The intention was for all trauma surgeons (orthopaedic surgeons), abdominal surgeons, and other senior doctors involved with trauma care (anaesthesiologists and radiologists) to complete the course.

Organizers from the two surgical departments (orthopaedic and abdominal) were given a list of available days and scheduled their surgeons when they were not occupied with other work tasks.

Each Monday, one to three participants were trained in the procedure on corpses before autopsies were done. Every surgeon had individual hands-on training and performed the procedure themselves. Before the course, references [5-8] were handed out to the participants.

**Figure 1:**
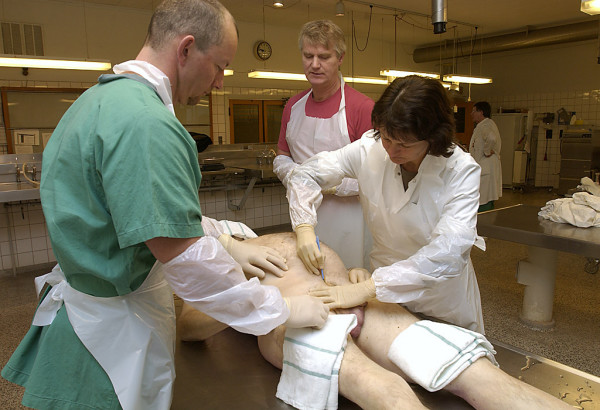
**Retroperitoneal packing is performed on a human corpse at the Department of Pathology**. A midline incision from the umbilicus to the symphysis is made. The abdominal musculature is divided until the peritoneum is reached. From here, it is possible to manually dissect the retroperitoneal space down into the pelvic space along the pelvic bones.

**Figure 2:**
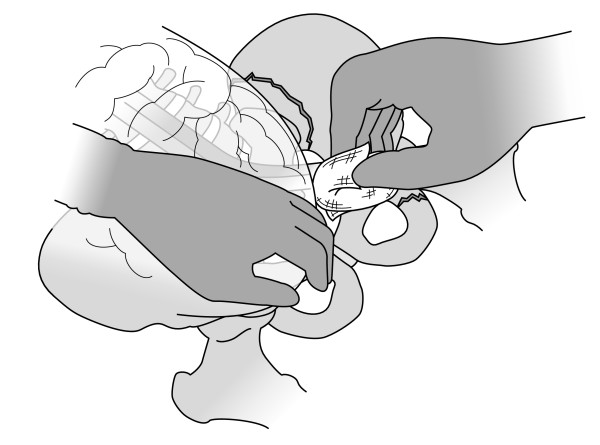
**Access to the retroperitoneal space in the left pelvic area is made**. The left hand pushes the peritoneum and intestines medial and cranial. Swabs are placed into the newly created space with the right hand. In a living patient, a haematoma would have dissected this space, which is then filled with swabs after the coagulum is removed.

**Figure 3:**
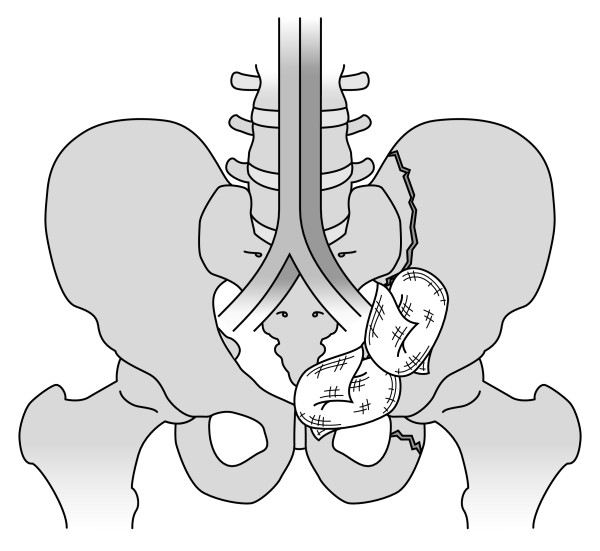
**Two or more swabs are placed to pack the left pelvic space**. The same procedure is used on the right side. Bilateral packing can be done in one to two minutes.

At the beginning of the session, the teacher briefly described the indications for the procedure, with emphasis on crisis management skills (Fig. 1). Next, the participants performed the procedure themselves. A simple midline incision from the umbilicus to the symphysis was made without opening the peritoneum. It was now possible to manually dissect down bilaterally on the inside of the pelvis, one side at a time, while the peritoneum and intestines were pushed upwards into the abdomen (Fig. 2). With this approach, it was possible to reach further down into the pelvis to os coccyx, and, in a matter of seconds, pack two or three swabs in each side (Fig. 3).

Afterwards, the subsequent decision management and ongoing treatment were discussed. The duration of the whole session was half an hour, and afterwards the corpses could undergo autopsy.

## Results

Twenty orthopaedic surgeons, 22 abdominal surgeons, two anaesthesiologists, and two radiologists from our own trauma centre together with two abdominal and one orthopaedic surgeon from other centres participated in the course. Four operation room nurses participated as observers.

One training session was cancelled because no surgeons enrolled for that specific Monday. Another three sessions were rescheduled because no corpses were booked for autopsy. The duration of the session never exceeded 30 minutes.

By the end of the course, all participants expressed that they had mastered the peritoneal packing procedure. More importantly, they felt comfortable making the decision to perform the procedure without hesitation when needed.

## Discussion

Trauma team training is an invaluable part of trauma care in any trauma organisation. The retroperitoneal packing training sessions have aided in developing professional multidisciplinary teamwork in real trauma situations. Emphasis has been placed on the importance on clearly communicating the background of broadly accepted guidelines [9]. However, some specific surgical procedures need to be taught either in real situations or on corpses.

Clinical research is an important factor in improving survival after critical incidences; however, it cannot stand alone. A new concept, 'Formula of Survival', has emphasised the importance of education and implementation of new knowledge into clinical practise [2,3]. Therefore, education and implementation have been a focus for developing our trauma organisation.

We have reported our initial results with this new surgical approach performed on patients with uncontrollable bleeding caused by pelvic fractures [5]. Before the course, packing had only been applied in two cases, but was indicated in a number of cases where it was not performed. During the first year (2007) after the course, packing was performed 11 times. Trauma audits after the course have shown that each packing procedure was performed correctly and without hesitation. Furthermore, there have been no cases where packing was indicated but not performed.

Penninga et al. [6] described the damage control concept and discussed, in a literature review, indications for damage control surgery.

Besides the ordinary Airway-Breathing-Circulation (ABC) approach, a correctly placed pelvic C-clamp is an obligatory part of the initial resuscitation of the majority of patients with pelvic fractures and bleeding complications [7].

To date, there are no randomized studies that report the value of damage control surgery including retroperitoneal packing. Still, most non-randomized studies and clinical guidelines support damage control surgery. Retroperitoneal packing is being increasingly recommended as a lifesaving procedure to be used in the hyper acute phase where the patient with pelvic fracture and severe uncontrollable bleeding is highly unstable [7,8,10,11].

Prior to the course, trauma audits in our centre have shown that surgeons are hesitant to perform the procedure. The surgeons knew that retroperitoneal packing was needed, but they lacked the confidence necessary to perform a procedure they had never tried, and perhaps never even seen before. This hesitation has resulted in patients dying due to bleeding shock when they were transferred to the CT scanner.

Our audits revealed that some patient deaths were a consequence of this hesitation; thus, we decided to train all trauma surgeons in retroperitoneal packing. After this course, our entire organisation was prepared to perform damage control surgery. Within 12 months of the course, lifesaving retroperitoneal packing was indicated and performed in 11 situations.

Angiographic embolisation is a recommended method to acquire haemostasis in arterial bleeding [12], but it is a time-consuming procedure where the patient has to be transferred to a radiographic department with specialised equipment. Furthermore, this service is rarely available 24 hours a day and is potentially unavailable altogether at smaller hospitals.

In our organisation, embolisation is not available 24 hours. It takes a long time to train the radiologists, and this service is expensive. However, embolisation is scheduled to be an integrated part of our trauma centre in the future.

The type of bleeding (arterial bleeding, venous bleeding, bleeding from bones and ligaments) responsible for hypovolemic shock and patient death is still being discussed. A Huittinens dissection study from 1973 examined 27 dead pelvic trauma patients and showed that all four sources of bleeding could be lethal [13]. Therefore, arterial embolisation alone cannot treat severe bleeding from pelvic trauma.

Unlike embolisation, we have shown that retroperitoneal packing can be taught to all surgical members of a trauma organisation in 6 months. Additionally, this was done without interfering with their daily duties.

Arterial embolisation and retroperitoneal packing complement each other. The priority of each procedure depends on the local setting. In a hyper acute situation, we would not recommend waiting for arterial embolisation, but we would quickly decide on and perform retroperitoneal packing.

Our course continues ad hoc as new doctors are hired to work in our trauma centre, and our operation room nurses are on a waiting list for the course. We are offering training on the retroperitoneal packing procedure to other hospitals in our region, so trauma patients with pelvic fractures and uncontrollable bleeding can be packed at their local hospital and be stabilised before being transferred to our trauma centre.

## Competing interests

The authors declare that they have no competing interests.

## Authors' contributions

AB drafted the manuscript, participated in the litterateur search, and in data interpretation. JB directed the retroperitoneal packing course, participated in the litterateur search, revised the manuscript, and participated in data collection and interpretation. KH is head of the orthopedic trauma section, revised the manuscript, and participated in data collection and interpretation. EF was head of the trauma centre, revised the manuscript, and participated in data collection and interpretation. All authors read and approved the final manuscript.
